# Postural control patterns in gravid women—A systematic review

**DOI:** 10.1371/journal.pone.0312868

**Published:** 2024-12-27

**Authors:** Wanda Forczek-Karkosz, Agata Masłoń

**Affiliations:** 1 Section of Biomechanics, Faculty of Physical Education and Sport, University of Physical Education, Krakow, Poland; 2 Section of Rehabilitation in Orthopaedics, Clinical Rehabilitation Institute, Faculty of Motor Rehabilitation, University of Physical Education, Krakow, Poland; West Virginia University, UNITED STATES OF AMERICA

## Abstract

**Background:**

Postural stability is essential for functional independence in the pregnant population. The contradictions between existing studies and the lack of consistent characteristics in the strategies used by pregnant women for postural control demonstrate the need for further investigation.

**Objectives:**

The aim was to review the available literature on postural strategies throughout pregnancy in both static and dynamic conditions and to provide an assessment of the quality of these studies in terms of methodological issues to identify the reasons for the inconsistencies in findings between research centers.

**Methods:**

Literature searches were conducted using PubMed and EBSCOhost Research Databases. The latest search was performed on September 01, 2024. The review was restricted to longitudinal, cross-sectional, case-control, and descriptive studies focused on the effect of pregnancy on the stability of future mothers, with the following criteria: healthy pregnant women and singleton pregnancies. Trials were excluded if they were restricted to multiple pregnancies or considered various kinds of interventions. The methodological quality was evaluated using the criteria proposed by Downs and Black. Data items such as information on study design, characteristics of the study sample, equipment used, stability task performance, and outcome measures were presented.

**Results:**

The final analysis comprised 22 articles, including a total of 641 pregnant and 296 nonpregnant women. Research results in both static and dynamic conditions are inconclusive, showing either a decrease, no change, or improvement in postural equilibrium as pregnancy advances. Importantly, the results indicate that women in advanced pregnancy may be at increased risk of falling when their vision is compromised.

**Discussion:**

A lack of homogeneity in the study groups and a small number of longitudinal analyses were observed. The methodologies applied and the postural indices used to measure body sway varied across the studies. Our findings can serve as basic data for health promotion programs to encourage safe daily activities in pregnant women.

## 1. Introduction

Postural control is a fundamental ability that not only provides the basis for standing and walking independently but also facilitates the performance of manual tasks. An erect bipedal position is regulated by sensory inputs (visual, vestibular, and somatosensory) to maintain postural equilibrium and proper alignment of body segments with respect to gravity [1–3]. Joint configuration, the center of mass (COM) position, and balanced muscles all contribute to optimal postural alignment. Understanding the motion of the COM with respect to the base of support (BOS) offers insights into balance control strategies [4, 5]. In static posturography, COM oscillations are represented by the center of foot pressure (COP) displacements [6]; thus, these measures are mostly used for postural balance assessment. However, increasingly more scientific reports emphasize that the goals of postural stability are broader than maintaining the COM above the base of support [7].

**Stability** is often described as being **static** or **dynamic**. Static stability is defined as the ability to minimize movement of the center of gravity within the base of support under a given condition [8]. Defining dynamic postural stability is more challenging. It is the ability to transfer the vertical projection of the center of gravity around the supporting base [9]. It has been measured following a perturbation of the support surface [10], a perturbation of the individual [11], or by requesting the individual to maintain balance following a change in position or location [12, 13]. There are two main processes used to restore or maintain postural stability: anticipatory postural adjustments (APAs) and compensatory postural adjustments (CPAs). APAs occur prior to movements and are a feedforward process that counteracts an expected perturbation [14]. They are considered the first line of defense for postural stability in anticipation of perturbations and have been observed while sitting, standing, or walking [15]. In contrast, CPAs are responses to external postural perturbations and are therefore under the control of feedback mechanisms [14].

Scientists point out that in everyday life, postural stability must be controlled in such a way as to ensure both the control of posture and the completion of various tasks (e.g., reading, talking, lifting an object). Research shows that performing more demanding tasks (e.g., reading) may result in a greater reduction in sway magnitude than accomplishing less demanding tasks (e.g., looking at a blank target) [16–18]. Given the complexity of maintaining postural stability, assessments should not only measure the magnitude of sway but also the ability to modulate it depending on the task, environmental, or individually variable factors.

One factor that may undoubtedly influence the need for adaptation to maintain balance and optimize joint load distribution is pregnancy. Typical pregnancy-related adaptations include a profound increase in body mass, primarily in the breasts and abdomen, fluid retention, and connective tissue laxity caused by hormonal changes [19–21]. These adaptations are followed by an increased anterior pelvic tilt [22, 23], increased lumbar lordosis [24, 25], and spine extension [26] to prevent the COM from shifting. The increased lumbar lordosis helps maintain an unchanged anterior-posterior position of the COM as pregnancy progresses [26]. Lateral stability, however, may be preserved during pregnancy due to an adaptive increase in stance width [23, 27].

The analysis and interpretation of postural sway should be carried out carefully because the data processing technique may affect the structure of COP variability [28]. Some authors emphasize deterioration in postural stability in the pregnant population [e.g., 29, 30]. Furthermore, some identified pregnant women as more prone to falls [31], which significantly increases adverse outcomes for mothers [21]. However, it is also proven that stability during pregnancy remains unchanged [32]. The aforementioned contradictions between existing studies and the lack of consistent characteristics in the strategies used by pregnant women for postural control demonstrate the need for further investigation into postural adaptations during pregnancy in terms of stability issues. It is of great importance to make women more aware of the alterations responsible for many discomforts in different body positions and during various activities.

### 1.1. Objectives

The aim of this paper is twofold: first, to review the available literature on postural strategies throughout pregnancy in both static and dynamic conditions; and second, to assess the quality of the studies in terms of methodological issues to identify the reasons for the inconsistencies in research findings on postural control in pregnant women.

## 2. Methods

This systematic review was conducted according to the Preferred Reporting Items for Systematic Reviews and Meta-Analyses (PRISMA 2020) guidelines [33]. A review protocol was not prepared.

### 2.1. Eligibility criteria, information sources, and search strategy

**Fig 1:**
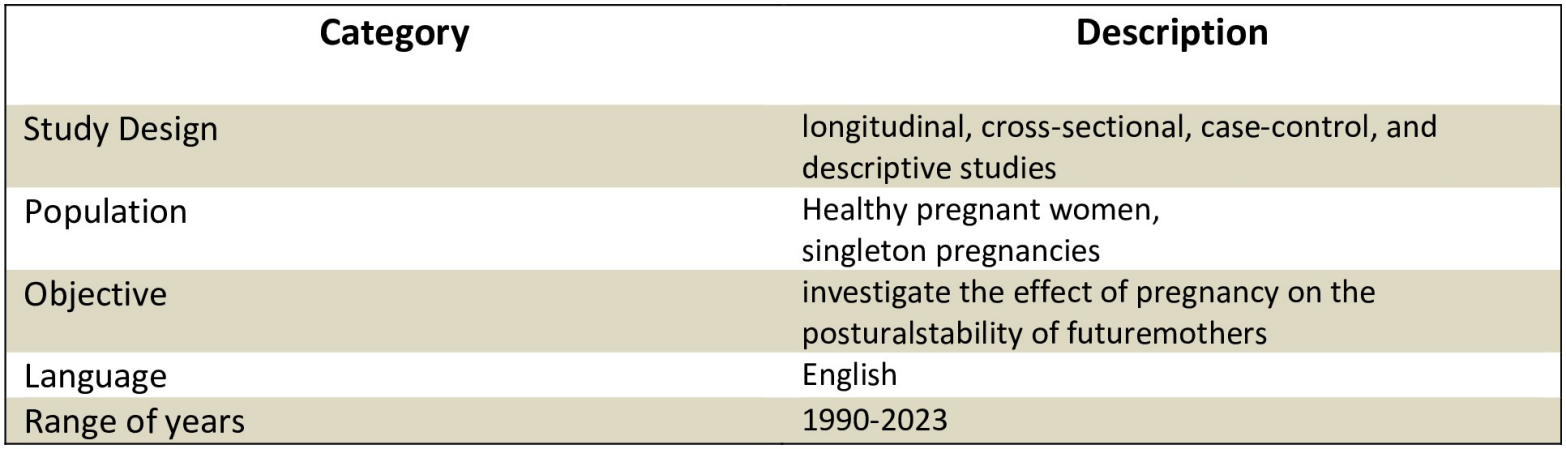
Inclusion criteria for systematic review of studies.

The inclusion criteria established by the authors were peer-reviewed papers published between 1990 and 2023 in English. The review was restricted to longitudinal, cross-sectional, case-control, and descriptive studies. Eligible studies should investigate the effect of pregnancy on the postural stability of future mothers with the following criteria: healthy pregnant women and singleton pregnancies. Trials were excluded if they met any of the following criteria: restricted to multiple pregnancies or considered various kinds of interventions (see Fig 1). Systematic reviews and meta-analyses were only included as background. Conference proceedings, letters, commentaries, editorials, abstracts only, or case studies were also excluded.

The main literature searches were conducted using PubMed and EBSCOhost Research Databases: MEDLINE, SPORTDiscus with Full Text, Rehabilitation & Sports Medicine Source, Health Source—Consumer Edition and Health Source: Nursing/Academic Edition. These databases were selected because of their broad inclusion of multidisciplinary topics within the Biomedical and Health Sciences domain. Each database was searched including the same range of years. The search strategy clustered terms used to describe studies investigating the effect of pregnancy on the postural stability of pregnant women (see S1–S3 Tables). The following search terms were used: “postural control” OR “postural balance” OR “balance” OR “postural stability” OR “body balance” AND (“pregnancy” OR “pregnant”). The restrictions applied to date and language: only English language studies published within 1990-2023were included. The latest search was performed on September 01, 2024.

### 2.2. Selection and collection of data

Two independent reviewers (W.F.K. and A.M.) analyzed the retrieved articles, taking into account the eligibility criteria. Initially, the papers were screened by checking the titles. Duplications were identified within the papers found, which included both duplicates between the databases and internal duplicates of the same research being published in more than one format. After removing duplicate publications each of the two reviewers read the abstracts of all articles, selected relevant articles according to the inclusion and exclusion criteria, and defined a list of articles for full-text reading. In case of disagreement, consensus on which articles to screen full-text was reached by discussion. Then, the researchers independently screened full-text articles for inclusion. In case of disagreement, consensus was reached on article inclusion or exclusion by discussion. Each reviewer independently reviewed each article to search for the data items. Data were extracted from article texts, tables, and figures.Some of the important information were copied and pasted into tables (e.g. technical details of the equipments used during the experiments), but the results were carefully selected and placed in tables in summary version.Afterwards, the extracted data were compared, and for any disparities, both reviewers determined the best-suited set of data through discussion.

### 2.3. Data items

For this systematic review, the following data items were presented: authors, title of the paper, year of publication, study design (Table 2). Other data items concerned subjects’ characteristics such as age, gestational week, parity, and sample size of the study (Table 3), as well as the objective of the study, outcome measures, the experimental setup regarding equipment used and tests performed, and the results of the study (Table 5).

### 2.4. Study quality assessment and synthesis methods

Due to key differences in the comparisons performed in each study and various outcome measures, we could not perform a meta-analysis of the included studies. Instead, we narratively synthesized the evidence.

The study quality was assessed using a modified checklist by Downs and Black [34] (Table 1). Because the included studies did not concern interventions, all questions in the Downs and Black quality assessment instrument that referred to interventions were omitted (14 questions were removed). Thus each article could score up to 13 points. Two reviewers assessed each study independently, and then assessment scores were compared and discussed in cases of disagreements until consensus was made. Table 2 provides the scores for all studies included in this systematic review. Each article could score up to 13 points. The authors recognized an article as of sufficient quality if it scored at least 6 points (representing a value of 50% + 1 point).

**Table 1:** Modified downs and black quality assessment checklist.

Reporting
**1.** Is the hypothesis/aim/objective of the study clearly described?
**2**. Are the main outcomes to be measured clearly described in the Introduction or Methods section?
**3.** Are the characteristics of the patients included in the study clearly described?
**6.** Are the main findings of the study clearly described?
**7.** Does the study provide estimates of the random variability in the data for the main outcomes?
**9.** Have the characteristics of patients lost to follow-up been described?
**10.** Have actual probability values been reported (e.g.0.035 rather than<0.05) for the main outcomes except where the probability value is less than 0.001?
External validity
**12.** Were the subjects asked to participate in the study representative of the entire population from which they were recruited?
**13.**Were the staff, places, and facilities where the patients were treated, representative of the treatment the majority of patients receive?
Internal validity–bias
**16.** If any of the results of the study were based on “data dredging”, was this made clear?
**17.** In trials and cohort studies, do the analyses adjust for different lengths of follow-up of patients, or in case-control studies, is the time period between the intervention and outcome the same for cases and controls?
**18.**Were the statistical tests used to assess the main outcomes appropriate?
Internal validity–confounding (selection bias)
**26.** Were losses of patients to follow-up taken into account?

**Table 2:** Design of the 22 studies on the effect of pregnancy on postural control and the results of their quality assessment.

Authors	Title	Year	Study design	Study quality
Butler EE, Colón I, Druzin ML, Rose J [29]	Postural equilibrium during pregnancy: decreased stability with an increased reliance on visual cues	2006	longitudinal and cross-sectional	9
Ribas SI, Guirro E [52]	Analysis of plantar pressure and postural balance during different phases of pregnancy	2007	cross-sectional	8
Jang., Hsiao KT, Hsiao-Wecksler ET [27]	Balance (perceived and actual) and preferred stance width during pregnancy	2008	longitudinal	11
Nagai M, Isida M, Saitoh J, Hirata Y, Natori H, Wada M [35]	Characteristics of the control of standing posture during pregnancy	2009	cross-sectional	-8
Oliveira LF, Vieira TM, Macedo AR, Simpson DM, Nadal J [32]	Postural sway changes during pregnancy: a descriptive study using stabilometry	2009	descriptive	-9
McCrory JL, Chambers AJ, Daftary A, Redfern MS [30]	Dynamic postural stability during advancing pregnancy	2010	longitudinal and cross-sectional	-13
McCrory JL, Chambers AJ, Daftary A, Redfern MS [42]	Dynamic postural stability in pregnant fallers and non-fallers	2010	longitudinal and cross-sectional	13
Moccellin AS, Driusso P [40]	Adjustments in static and dynamic postural control during pregnancy and their relationship with quality of life: A descriptive study	2012	descriptive	13
Yu Y, Chung HC, Hemingway L, Stoffregen TA [49]	Standing body sway in women with and without morning sickness in pregnancy	2013	retrospective	-9
Inanir A, Cakmak B, Hisim Y, Demirturk F [41]	Evaluation of postural equilibrium and fall risk during pregnancy	2014	cross-sectional	10
Ersal T, McCrory JL, Sienko KH [31]	Theoretical and experimental indicators of falls during pregnancy as assessed by postural perturbations	014	retrospective	-9
Takeda K, Shimizu K, Imura M [45]	Changes in balance strategy in the third trimester	2015	longitudinal	-8
Yoo H, Shin D, Song C [36]	Changes in the spinal curvature, degree of pain, balance ability, and gait ability according to pregnancy period in pregnant and nonpregnant women	2015	single-blinded cross-sectional	10
Opala-Berdzik A, Błaszczyk JW, Bacik B, Cieślińska-Świder J, Świder D, Sobota G, Markiewicz A [48]	Static Postural Stability in Women during and after Pregnancy: A Prospective Longitudinal Study	2015	longitudinal	13
El-Shamy FF, Ghait AS, Morsy M [47]	Evaluation of Postural Stability in Pregnant Women	2016	case control	11
Moreira LS, Elias LA, Gomide AB, Vieira MF, Do Amaral WN [37]	A longitudinal assessment of myoelectric activity, postural sway, and low-back pain during pregnancy	2017	longitudinal	12
Opala-Berdzik A, Błaszczyk JW, Świder D, Cieślińska-Świder J [50]	Trunk forward flexion mobility in reference to postural sway in women after delivery: A prospective longitudinal comparison between early pregnancy and 2- and 6-month postpartum follow-ups	2018	longitudinal	11
Danna-Dos-Santos A, Magalhães AT, Silva BA, Duarte BS, Barros GL, Silva MFC, Silva CS, Mohapatra S, Degani AM, Cardoso VS [38]	Upright balance control strategies during pregnancy	2018	cross-sectional	8
Takeda K, Yoshikata H, Imura M [46]	Changes in Posture Control of Women That Fall During Pregnancy	2018	longitudinal	9
Shingala RK, Desai M, Honkalas P, Kumar A [39]	Evaluation of postural balance in third trimester Pregnancy	2019	cross sectional observational	9
Sancar S, Guzel NA, Cobanoglu G, Ozdemir YA, Bayram M [44]	The Changes in Static Balance During Pregnancy: A Prospective Longitudinal Study	2021	prospective longitudinal study	12
Ramachandra P, Kumar P, Bø K, Maiya A [51]	Comparison of static postural sway characteristics between pregnant andnon-pregnant women	2023	cross-sectional comparison study	12

*Study quality assessment was performed using a modified Downs and Black Quality Assessment Checklist

## 3. Results

### 3.1. Study selection

**Fig 2:**
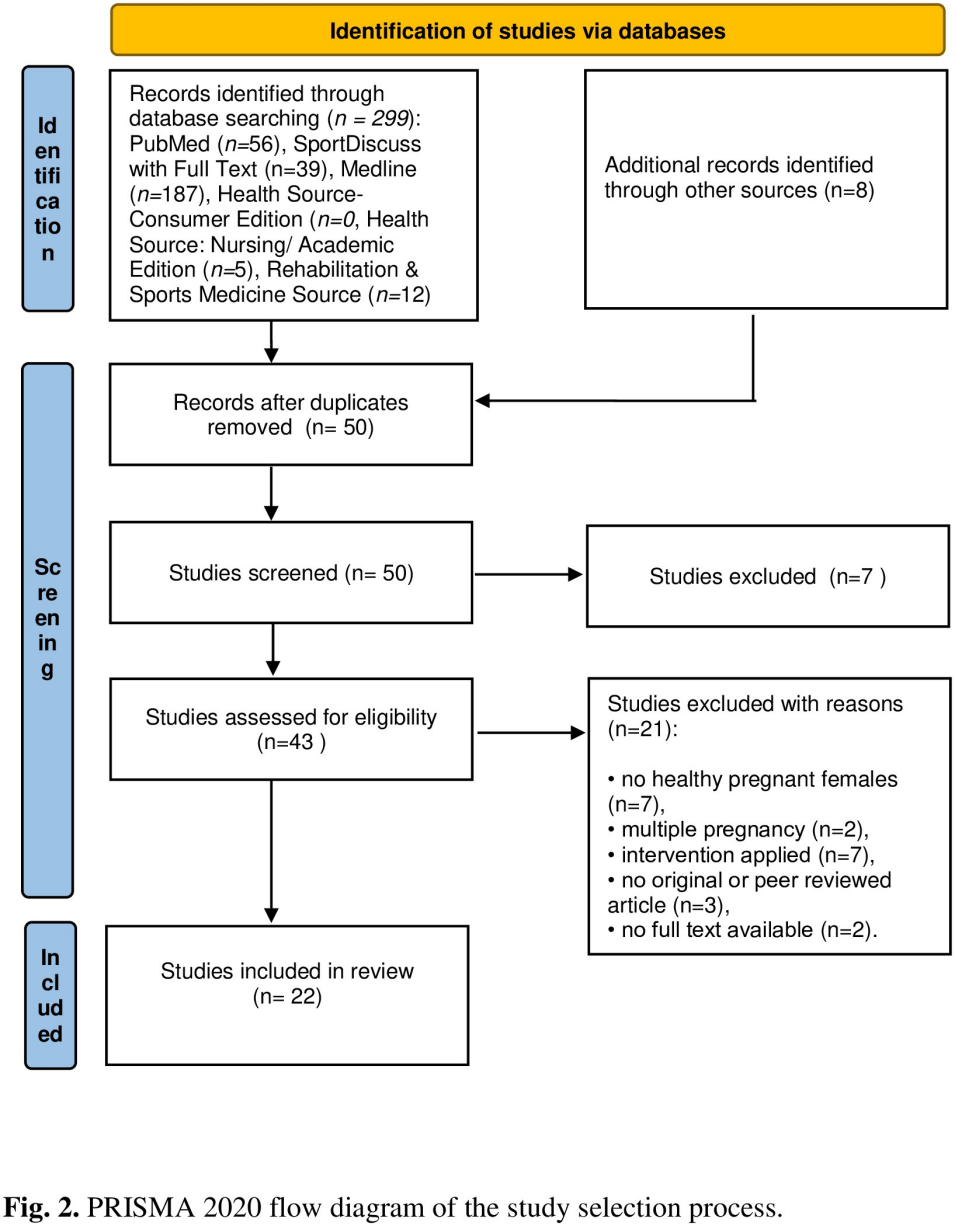
PRISMA flow chart.

After screening the titles and abstracts, from a total 243records from EBSCOhost, only 35 remained for the analysis, while from a total 56 PubMed records, only 7 remained for the analysis. Additionally, through other sources, we identified 8 papers. After full-text assessment, 22 studies were included in this systematic review. The process of study selection in this review is described in the PRISMA flow diagram (Fig 2), along with the reasons for exclusion.

### 3.2. Study characteristics

#### 3.2.1. Study design

The studies are presented in chronological order based on the publication year (Table 2). Ten of the studies were longitudinal, seven were cross-sectional, two were descriptive, two were retrospective, and one was a case-control study. Among these qualified articles, 14 focused on static stability, four on dynamic stability, and the remaining considered both. Considering the countries where the studies were conducted, seven were carried out in the USA [27, 29, 30, 38, 42, 43, 49], four in Brazil [32, 37, 40, 52], and three in Japan [35, 45, 46]. Other countries, like India [39, 51], Turkey [41, 44], and Poland [48, 50], each had two studies on this issue. Finally, investigators from the Republic of Korea [36] and Egypt [47] provided one paper each.

#### 3.2.2. Group characteristics

Among the analyzed studies, 15 (out of 22) included control groups (Table 3). The mean number of participants for the pregnant group was 28.8 ± 20.7, and for the nonpregnant control group, it was 21.1 ± 12.3. The mean age of the pregnant women included in the analyzed studies was 28.8 years, whereas the mean age of the control group was 26.9 years.

**Table 3:** Characteristics of the study sample of the included studies on the effect of pregnancy on postural control.

References	Subjects’ age [yrs]	Parity	Gestionalage	Samplesize
**Butler et al. (2006)** [29]	P:32.8 ±5 NP:31.2 ±6	P: 11 primigravid + 1 multigravida NP: nulligravid	1T: 11–14 w.p. 2T: 19–22 w.p. 3T: 36–39 w.p. PP: 6–8 w. pp.	n = 12 P n = 12 NP
**Ribas&Guirro (2007)** [52]	P: 1T: 24±6.32 2T: 25.6±7.5 3T: 21.6±4.3 NP: 22±1.21	No information	1T: up to 12 w.p. 2T: between 13–24 w.p. 3T: upwards of 25 w.p.	Initially: n = 72 Finally: n = 60 n = 45 P (15 subjects in each trim. (1–3) n = 15 NP
**Jang et al. (2008)** [27]	P: 25–38, NP: 24–39, [start of testing 31 ± 4yrs (bothgroups)]	P: 5 primigravid NP: no information	P: by 16 w.p., -the 4-week intervals until delivery, PP: 6 w. pp. 12 w. pp. 6 months pp. NP: • at 4-week intervals for 40 weeks, • then 6 weeks, 12 weeks and 6 months after 40^th^week	Initially: n = 15 P n = 15 NP Finally: n = 12 P n = 13 NP
**Nagai et al. (2009)** [35]	P: 30.6±0.6 NP: 35.4±3.1	No information	1 session of P (30.3±0.8 w.p) divided into: HA- a high anxiety group LA -a lowanxiety group	n = 35 P n = 8 NP
**Oliveira et al. (2009)** [32]	28.7±6.2	No information	1T: 15.1 ±1.8 w.p. 2T: 24.0 ±2.4 w.p. 3T: 34.5 ±2.5 w.p.	n = 20 P
**McCrory et al. (2010)** ^**a**^ [30]	P: 29.5 ± 4.9 NP: 26.5 ± 6.4	No information	2T: 20.9 ±1.2 w.p. 3T: 35.8 ±1.5 w.p. NP data were collected in the week following menses	n = 81 (41 P + 40 NP) [2T n = 41, 3T n = 29].
**McCrory et al. (2010)** ^**b**^ [42]	P: 29.5 ± 4.9 NP: 26.5 ± 6.4	No information	2T: 20.9 ±1.2 w.p. 3T: 35.8 ±1.5 w.p. NP data were collected in the week following menses	n = 81 (41 P + 40 NP) [2T n = 41, 3T n = 29].
**Mocellin&Driusso (2012)** [40]	P: 1. 29.15± 5.64 2. 29.23± 5.79 3. 29.46± 5.83 NP: 26.07 ± 3.98	P: no information NP: nulligravid	1T: 10–14 w.p. 2T: 22–24 w.p. 3T: 32–34 w.p. + NP	Initially n = 24 P Finally n = 13 P + 20NP
**Yu et al. (2013)** [49]	P (Wellgroup): 30.5±3.1 P (Sick group): 29.9±3.0	P: 10 primigravid and 11 multigravid	T1 Sick group (≥8 in Rhodes index of nausea, vomiting and retching) Well group	n = 21 P n Wellgroup = 9, n Sick group = 12
**Inanir et al. (2014)** [41]	P: 1T: 26.1 ± 4.9 2T: 26.2 ± 5.2 3T: 26.4 ± 5.8 NP: 23.1 ± 5.0	P: 1T (parity 1.2 ± 0.9, gravidity 2.3 ± 1.0), 2T (parity 1.0 ± 0.9, gravidity 2.0 ± 1.0), 3T (parity 1.0 ± 0.8, gravidity 2.1 ± 0.9) NP: (parity 1.6 ± 0.8, gravidity 1.7 ± 0.9)		n = 80 P including: 1T = 25 2T = 30 3T = 25 n = 30 NP
**Ersal et al. (2014)** [43]	P-fallers: 29.4±4.7 P-non-fallers: 30.6±3.8 NP: 26.5±6.4	P: 27 primigravid; 5 –secondpregnancy, 9—third pregnancy NP: 33 nulligravid, 6—pregnant one time and 1 –pregnanttwice	2T: 20.9±1.2 w.p. 3T: 35.8±1.5 w.p. NP—single data collection session.	Initially: n = 41 P n = 40 NP Finally: n = 29 P (15 women with a fall history during pregnancy, 14 women without a fall history during pregnancy), n = 40 NP
**Takeda et al. (2015)** [45]	P: 28.3±3.4 NP: 21.3 ±0.9	No information	2T 3T NP: single data collectionsession.	n = 8 P n = 8 NP
**Yoo et al. (2015)** [36]	P: 29.54±3.45 NP: 8.85±3.02	No information	2T: 26 ±1.67 w.p. 3T (no informationabout w.p.)	Initially: n = 19 P (2T), n = 15 NP Finally: n = 16 P (3T), n = 15 NP
**Opala et al. (2015)** [48]	28.2±3.6 (20–38)	P: 26 primigravid 5 multigravid	P: 1T: up to 16 w.p. 3T: ~3 weeks before due date; PP: ~2 m. pp. 6 m. pp.	Initially: n = 45 P Finally n = 31 P
**El-Shamy et al. (2016)** [47]	27.02±1.2	P: Primigravid	2T: 20–24 w.p. 3T: 30–34 w.p.	Initially: n = 31 P Finally: n = 14
**Moreira et. al. (2017)** [37]	P: 25.8 ± 4.6 NP: 27.7±5.5	P: Primigravid NP: Primigravid	1T: 10–14 w.p. 3T: 30–33 w.p.	n = 15 P + 15 NP
**Opala et al. (2018)** [50]	28.6±4.4 (20–38)	P: 13 primigravid + 4 multigravid	P: 1T: 7–12 w.p. PP: 2 m. pp. (6–10 w. pp) 6 m. pp. (25–28 w. pp)	N = 17 P
**Danna-Dos et al. (2018)** [38]	1T: 28 (21–30) 2T: 24.5 (22.2–27) 3T: 25 (23.5–29.5) NP: 23 (22–25)	P: no information NP: nulliparous	1T 2T 3T	n = 30 P Including: 1T, 2T, 3T of 10 P each n = 10 NP
**Takeda et al. (2018)** [46]	32 ± 3.2 (20–30)	Fall group: 2 primigravid 8 multigravid Non fall group: no information	2T 3T no info about w.p.	Initially: n = 100 P Finally: n = 82 P Non fall group n = 72 Fall group n = 10
**Shingala et al. (2019)** [39]	20–30	No information	3T no info about w.p.	n = 60 (30 P + 30 NP)
**Sancar et al. (2021)** [44]	29.31 ± 5.57	No information	P: 1T: 10–12 w.p. 2T: 22–24 w.p. 3T: 34 w.p.- delivery	n = 19 P
**Ramachandra et al. (2023)** [51]	P: 25.4 ± 3.1 NP: 24.4 ± 4.0 (18–30)	P: Primigravid	3T: 32 w.p.	Initially: n = 68 P, n = 52 NP Finally: n = 40 P, n = 40 NP

w.p.–week of pregnancy, 1,2,3T –first, second, and third trimester of pregnancy, pp–postpartum, P—pregnant women, NP–nonpregnant, CG–control group

In 12 out of 15 studies with a control group, one or more of the following factors were considered in matching the research and control groups: age [27, 29, 35–39], body mass [29, 35, 40, 41], BMI [29, 30, 35, 40–43], and body height [29, 35, 36, 40]. Considering age and body height, no significant differences between control and study groups were observed. However, in four studies, control and study groups varied significantly in body mass and BMI. In two of them, this was more understandable since it was observed for women in late pregnancy [35, 41], while in the remaining two, significant differences in either body mass [40] or BMI [38] were observed even for women in early pregnancy. In 14 studies, information about parity was included. Among those including nonpregnant control groups, some provided information about parity for both pregnant and nonpregnant groups [29, 41, 43], while others provided it either for the nonpregnant [38, 40] or pregnant [27, 37] group. One study included figures on both parity and gravidity and showed that the groups were matched for these factors [41]. All pregnancies of the women included in the analyzed studies were singleton. The longitudinal analysis of postural stability throughout the three trimesters of pregnancy was shown in five studies [27, 29, 32, 40, 44], while eight studies analyzed two trimesters (2nd and 3rd [30, 36, 42, 43, 45–47] or 1st and 3rd [37, 48]). Four studies included women in either the 1st [49, 50] or 3rd [35, 39, 51] trimester of pregnancy. Furthermore, three studies provided an analysis of postural stability across all three trimesters; however, this was not a longitudinal analysis as the group was tested once and consisted of women at different stages of pregnancy [38, 41, 52]. Four of the analyzed studies assessed postural stability postpartum, mainly considering the first six months after delivery. The number of postpartum experimental sessions ranged from one (6–8 weeks postpartum [29]), through two (2 and 6 months postpartum [48, 50]), up to three (6 weeks, 12 weeks, and 6 months postpartum [27]). In most studies, a single data collection session for the nonpregnant control group was planned, whereas in one study, the control group was tested using the same time scheme as the research group [27]. The division into trimesters varied greatly between the studies. Furthermore, in some studies, despite a division into trimesters, there was no information about the week of pregnancy when the data were collected [38, 39, 41, 46, 49].

### 3.3. Risk of bias in studies

The methodological quality concerning the risk of bias in the included studies was evaluated using the criteria proposed by Downs and Black [34]. These characteristics allowed for comparing articles, especially regarding their methodological design, sample size, measures of exposure, and definitions of outcomes. Because the included studies did not concern interventions, all questions in the Downs and Black quality assessment instrument that referred to interventions were omitted (14 questions were removed). Thirteen out of the possible 27 questions were used for quality assessment, as presented in Table 1.

Using the modified Downs and Black Checklist, the included studies scored between 8 [23, 26, 33, 39] and 13 [18, 30, 36] points for quality, out of a possible 13 points (S4 and S5 Tables). Categorization of total scores obtained allowed us to determine that analyzed studies were of moderate and strong quality (Table 4). All of the studies clearly described their objectives and main outcomes to be measured and used the appropriate statistical tests to assess the results. Several studies did not adequately describe patient characteristics and main outcomes including not providing estimates of the random variability in the data and actual probability values. In the vast majority of studies, recruitment of the study participants, the staff and facilities where the study was conducted as well as characteristic of patients lost to follow-up information have not been described.

**Table 4:** Categorization of total scores obtained using the modified downs and black checklist.

Quality Index*	Percentage	MethodologicalQualityScore	Number of articles
Strong	≥75%	≥10	12
Moderate	50–75%	7–9	10
Limited	25–49%	3–6	0
Poor	<25%	<3	0

*Adapted from Hartling et al. and Hignett; Out of a possible 13 points.

### 3.4. Results of individual studies

The methods used as well as the results of the studies, are presented in Table 5.

**Table 5:** Assessment methods and results of the studies on the effect of pregnancy on postural control.

Reference	Objective	Outcome measures	Equipment	Test performed	Results
**Butler et al. (2006) [29]**	to determine whether there are changes in postural equilibrium in pregnancy	**Static stability:** • average radial displacement (ARD) [cm] • velocity of path length (CoP distance) [cm/s]	**force platform** (50x 50 cm; model 9284; Kistler Instrument Corp, Amherst, NY)	**Body position and task:** quiet standing on a force platform with eyes open (EO) and closed (EC). **Number of trials:** 3 for each condition (EO, EC) **Trial time:** 30 s	no sign. differences between CG and 1T, except for ARD in EC, path length/s and ARD sign. higher in 2 and 3T than CG in both EO and EC, sign. elevated balance measures in PP vs. CG, increased reliance on visual clues to keep balance as pregnancy progressed.
**Ribas&Guirro (2007) [52]**	to analyze postural balance during 3 trimesters of pregnancy to correlate postural balance during 3 trimesters of pregnancy with anthropometric characteristics	**Static stability:** • AP and ML center of force (COF) oscillations [mm] • distance from the COF to the anterior (COF-A) and posterior (COF-P) limit of the feet [cm]; • width of support base (LB) [cm]—distance between the medial edge of R and L foot **Anthropometry** Body mass [kg] Body height [m] BMI [kg/m^2^]	**Pressure Platform:** Computerized Baropodometry System–Pressure Platform–Matscan 5.1 (Tekscan) [40 Hz]	**Body position and task:** orthostatic position gazing at eye level with arms to the side and free support base **Number of trials:** 3 **Trial time:** 5 s	no sign. differences between the groups in COF-A and COF-P, sign. greater COF (AP) amplitude in 3T vs. 1T, no difference in COF (ML) amplitude, positive correlation between AP COF oscillation and the support base in 1T, sign. narrower LB in 2T vs CG, no correlation between COF oscillation and weight or weight gain in P
**Jang et al. (2008) [27]**	to track balance and stance width throughout pregnancy and postpartum	**Static stability:** Stabilogram traditional parameters: • SD_AP_, _ML_, _RAD_ combined radial directions [mm]; • Vel-mean sway velocity in 3 directions [mm/s]; • 95Freq- 95% power frequency in AP and RAD directions [Hz]; • AngDev- angular deviation of principal sway direction from AP axis [deg] Stabilogram diffusion analysis (SDA) parameters: • short-term and long-term diffusion coefficient (D_S_,D_L_) [mm^2^/s] • scalling exponents (H_S_, H_L_) in the AP, ML, RAD directions [mm^2^/s] **Preferred stance width(SW)–**distance between heels [cm]	**Force plate** (model BP600900, AMTI, Watertown, MA) [100 Hz] **Self-evaluation questionnaire**–to quantify perceived sense of balance	**Body position and task:** standing unshod with both feet on a force plate with arms at the side and eyes open, looking at the stationary picture placed at eye level and 3 m away. Self-selected stance width for each trial. **Number of trials:** 10 (short rest breaks in-between) **Trial time:** 10 s	sign. elevated balance measures in the radial and AP directions in P and their decrease in PP, average AP and RAD measures increased in P, but due to their large variability no sign. differences in 2 and 3T. sign. decrease in SD_AP_, Vel _AP_, D_SAP_ from 3T to PP; relatively stable ML sway in P but tended to increase in PP. sign. increase of Vel ML from 3T to PP; sign. increase of SW in P and dropped to control levels in PP; D_S AP_ correlated with SB and SW parameters.
**Nagai et al. (2009) [35]**	to clarify how the contribution of sensory inputs varies in response to physical and mental challenges during pregnancy	**Static stability:** • the path length [cm] and area [cm^2^] of body sway; • spectrum analysis by fast Fourier transform (FFT) method of body sway in ML and AP axes;	**Force platform** equipped with a data processor (Gravicorder G-5500, Anima, Tokyo, Japan)	**Body position and task:** standing on the platform with the feet parallel, gazing at a target fixed at a 1.5 m distance and at the height of participant’s eyes. **Number of trials:** 2: EO(1), EC(2) **Trial time:** 1min	sign. greater enveloped area of body sway in P vs NP. sign. increase of the total path length in P vs NP in EC, sign. increase of the ratio of T-LNG to ENV-AREA in EC, and sign. smaller in P vs NP, sign. increase of the path length in the ML axis (X-LNG) in EC and a sign. interaction between groups and conditions, no difference in the ML body sway between P and NP, Increase of the path length in the AP axis in EC and in P (no sign.) greater than that in NP (in both EO and EC).
**Oliveira et al. (2009) [32]**	to detect and analyze changes in body sways in both time and frequency domains, over the course of pregnancy accounting for possible effects of reducing the support base and suppressing the visual inputs.	**Static stability** • COP area (ML & AP deflections [mm^2^] • Total Power (TP) for AP & ML, to obtain a global measure of COP sways • BoS size [cm] (distance between the mid-points of the long axis of each foot (from hallux to the middle of the heel)	**Custom force plate** **[50 Hz]** developed in accordance with the specifications provided by the French Association of Posturology [23] and Bizzo et al. [1985].	**Body position and task** **No of trials:** 30 stabilometric tests in 4 conditions: 1. EO: feet comfortably apart (EO/FA); 2. EC: feet comfortably apart (EC/FA); 3. EO: feet together (EO/FT); 4. EC: feet together (EC/FT). **Trial time:** 2-minute rest periods between each test.	sign. increase of COP area in 2 and 3T vs early P except for EO/FA the highest TP for COP sways along AP in all protocols for 3T (sign. in EC/FA & EO/FT), sign. increase of TP of COP sways along ML in EC/FA in 2 and 3T vs early P, no sign. change of BoS size with pregnancy.
**McCrory et al. (2010a) [30]**	to investigate the effects of advancing pregnancy on dynamic postural stability	**Dynamic stability** COP metrics: sway responses: • initial sway [cm] • total sway [cm] • sway velocity [cm/s] • reaction time [ms]	**Equitest posture platform** (Neurocom, Int., Clackamas, OR, 100Hz) COP recordings were made during each perturbation response (small, medium, and large magnitude)	**Body position and task** Subjects staring straight ahead stood on the platform fitted in a chest and hip harness (MCT). **No of trials:** 3 trials	no differences in any measures 2T vs NP, sign. decrease in all sway responses in 3 vs 2T, reaction time is not affected by pregnancy.
**McCrory et al. (2010b) [42]**	to compare dynamic postural stability in PW who have fallen during pregnancies with those who have not and with NP	**Dynamic stability** COP metrics: sway responses: • initial sway [cm] • total sway [cm] • sway velocity [cm/s] • reaction time [ms]	**Equitest posture platform** (Neurocom, Int., Clackamas, OR, 100Hz) COP recordings were made during each perturbation response (small, medium, and large magnitude).	**Body position and task** See above	smaller COP sway responses in P vs NP, initial sway sign. less in PF vs PNF and NP, sway velocity and total sway sign. less in PF vs PNF and NP, sign. different sway velocity and total sway between the forward and backward perturbations, and between the small, medium, and large perturbations.
**Mocellin&Driusso (2012) [40]**	to analyze changes in static and dynamic postural control during 3 trimesters,	**Static stability** COP metrics: • COP areas in ML & AP directions [cm^2^] • mean values of 3 trials used for analysis **Dynamic stability** • GRF [% of b. mass]: • time and value of the first peak (P1), second peak (P2) and valley of the vertical component; time, max / min value in AP component;	**force plate** (Bertec^®^, 100 Hz) **2 force plates** (Bertec, Columbus, OH, USA)	**Body position and task** standing barefoot staring at an eye-level height point 2m away from the force plate: • bipedal posture with feet together (EO and EC) • bipedal posture with feet together and in tandem position with EO, alternating right leg (TRL) and left leg (TLL) in the front. **No of trials: 3** **Trial time:** 60 s **Task walking** over the platform, using the shoes and stare at an eyelevel height point located 2m away from the force plate. **No of trials:** 5	Static stability: • COP areas in T1 greater than NP in all positions, but no sign. difference between NP vs T1, or between T1, T2 and T3, • in T2: sign. negative correlation between COP area in the TLL position, Dynamic stability • GRF—different in NP than in P (not sign): • longer time in the first phase of weight acceptance, • lower 1^st^ and 2^nd^ peaks of vertical GRF, • lower max and min of A-P GRF. • the same pattern of GRF across 3 trim. of P
**Yu et al. (2013) [49]**	to assess whether morning sickness is associated with changes in standing postural equilibrium	**Static stability in different conditions (tasks):** Balance measures: positional variability of the COP in AP and ML axes [cm] Detrended fluctuation analysis (DFA): α—scaling exponent of DFA **Perceived balance stability (SB)**—from 0 (normal) to 10 (extremely unstable) **Experience of motion sickness during pregnancy** -question (yes/no) **Rhodes Index (RI)**—score 8 to 40	**Force platform:** (50 cm x 50 cm, AccuSwayPlus, AMTI, Watertown, MA, sampled at 50 Hz) Questions about perceived balance stability and experience of motion sickness **Rhodes index of nausea,** **vomiting, and retching**	**Body position:** Participants stood (in stocking feet) on a stable force plate with their heels on a line 1.0 m from the visual Target. 3 variations in stance width: with the heels 5 cm, 17 cm, or 30 cm apart. Stance angle was held constant. **Visual search task** (counting the number of times the target letter appeared in the block of text (1 of 4 blocks of English text each consisting of 13 or 14 lines of text) and also reporting their position in the text at the end of the trial) **Inspection task** (quiet standing on the platform with the gaze kept within the borders of the target (blank sheet of white paper)). **Number of trials:** one 60 s trial in each of 6 conditions (2 (visual task) x 3 (stance width). **Trial time:** 60 s each The order of conditions was randomized across participants.	decrease of positional variability with wider stance and reduction during more demanding visual task; sign. decrease of positional variability in AP axis during the Search task, relative to sway during the Inspection task. in AP axis, a sign. main effect of visual task on α. Movement was less self-similar during the Search task than during the Inspection task. in the ML axis, the main effects of stance width was sign., positional variability differed between each pair of conditions (5 cm > 17 cm > 30 cm). stance width x visual task interaction was also significant. in the ML axis, the main effect of stance width on α was sign. and differed between each pair of conditions (5 cm > 17 cm > 30 cm). sign. greater instability (SB) for the Sick group than the Well group. greater positional variability for the Well group than for the Sick group
**Inanir et al. (2014) [41]**	to evaluate dynamic postural stability and fall risk during pregnancy and compare dynamic postural stability between P and NP	**Dynamic stability:** • overall stability index: OA • AP stability index: APSI; • ML stability index: MLSI **The fall risk test (FRT)** evaluated by OA, APSI and MLSI.	**Balance platform:** Biodex Balance System (BBS) (Biodex Medical Systems, Shirley, NY, USA) movable balance platform: the system’s difficulty levels varied between 1 (most difficult) and 8 (the easiest).	**Body position and task:** Standing barefoot on the BBS platform (difficulty level 8) in a convenient position with knee flexed 15°, arms placed across the chest and glare fixed ahead. **Number of trials:** 3 **Trial time:** 20 s	no sign. differences for OA, APSI, MLSI and FRT among the PW in 1T, 2T and CG. sign. higher all postural stability and FRT scores in 3T vs CG sign. higher 3T indices for OA and MLSI than 1T, sign. higher FRT scores in 3T vs 1 & 2T
**Ersal et al. (2014) [43]**	to investigate whether COP–COG can differentiate pregnant fallers from nonfallers to use mathematical models to gain insights into the differences in postural control strategies between pregnant fallers and nonfallers	**Dynamic stability:** COP [cm] (measured directly) and COG [cm](estimated by the Equitest platform) Peak COP–COG [cm] Two-segmented mathematical model to represent the dynamics of the body.	**The Equitest platform** (NeuroCom International, Inc., Clackamas, OR, USA)	**Body position and task:** standing on the platform with the feet hip-width apart and stare straight ahead. All subjects were fitted with a chest and hip harness. MCT protocol; Translational surface perturbations in the AP directions generated by the platform. **No of trials:** 3 trials performed at small, medium, and large perturbation magnitudes	sign. smaller peak COP–COG values in P fallers vs P nonfallers and CG; perturbation magnitude was a sign. factor but perturbation direction was not sign increase of peak COP–COG from the small to medium perturbations, as well as from the medium to large perturbations acc. to the model: P nonfallers had increased ankle stiffness compared with P fallers and CG
**Takeda et al. (2015) [45]**	to analyze and clarify by kinetics the joint moment changes that affect balance during pregnancy	**Static stability:** • FRT max—the max FRT distance [cm], • COP anterior displacement at FRT max [cm], • GRF (vertical and anterior) at FRT max [N/kg],	**Two force plates** (AMTI MA, USA) **A Vicon Nexus 3D motion analysis system** (Vicon Peak Oxford, UK). **10 infrared cameras** (120 Hz) 15 reflective markers (25 mm) placed according to [Clinical Gait Analysis Forum of Japan, Gait data interface manual, proposal for the standardization of gait data format, 1st ed. Tokyo: Ishiyaku Publishers, 1992, pp 55–58].	**Body position and task:** barefoot static standing posture on the force plates with the feet shoulder-width apart, with both arms at sides, and eyes focused horizontally forward. From this static standing posture functional reach test (FRT) was performed **Number of trials:** 3	sign. smaller anterior COP displacement of L leg at FRT max in 2T and 3T vs NP; FRT max sign. smaller in 2 and 3T vs NP; sign. smaller posterior GRF of right leg in 2 and 3T vs NP; sign. larger vertical GRF of the L leg (2T) and both legs (3T) compared to NP; sign. larger posterior FRF and vertical FRF of the R leg in 3 vs 2T.
**Yoo et al. (2015) [36]**	to investigate the changes in balance and gait ability as well as pain intensity and spinal curvature throughout pregnancy vs NP	**Static stability** weight distribution index **(WDI)** **Dynamic stability** • gait parameters: velocity [cm/s] cadence [step/min]	**4 force plates** **Tetrax**^**®**^ **system** (Sunlight Medical Ltd., at Ram Gan, Israel). Each force plate measures perpendicular pressure of the anterior and posterior feet. **GAITRitesystem** (GAITRite GOLD, Version 3.2b, software (CIR System Inc., Sparta, NJ, USA, 2007),	**Body position and task** standing comfortably with EO / EC on a normal plate and flexible plate **No of trials:** 3 **Trial time:** 30 s **Body position and task** subjects walk 2 m before reaching and after leaving the mat at comfortable speed	• sign. decrease of WDI under all conditions in 3T vs 2T (improve of balance) • sign. decrease of balance in PW vs NP only on unstable surfaces Dynamic stability • sign. lower speed & cadence in 3 vs 2T • sign. lower speed in P vs NP
**Opala et al. (2015) [48]**	to compare static postural stability in women between early pregnancy, advanced pregnancy, and at 2 and 6 months pp. to investigate whether body mass, base of support width, and amount of sleep within 24h before testing might affect static stability of pregnant/ pp women	**Static stability** COP metrics: AP / ML path length [mm] AP / ML velocity [mm/s] stance width [mm] sleep duration [h] within 24h before testing **Anthropometry** Mass [kg] BMI [kg/m^2^]	**force plate** (model 9281C,Kistler Instruments Corp, Winterthur, Switzerland) (100 samples/s)	**Body position and task** barefoot quiet standing with arms at sides and at a preferred stance width on a stable force plate: • with EO (looking straight ahead at a wall 3m away) • with EC. Breaks, up to 1min, separated the trials. **No of trial:** 2 **Trial time:** 30 s	sign. increase of AP sway in EC in late P stance width weakly positively correlated with AP sway in EO; sign. weak positive correlation with COP AP- path length and mean velocity in EC; sign increase of BoS width from early to late P, decrease from late P to 2 m. PP, and remained decreased at 6 m PP; P/ PP body mass weakly positively correlated with AP sway in EC; no effect of sleep duration on postural sway
**El-Shamy et al. (2016)** **[47]**	to assess postural balance during 2 and 3 T of pregnancy	**Dynamic balance** stability indices: AP, ML and Overall (OA) of COP at level 7 and 8	**Biodex Balance System** (Inc, Shirley New York, USA)	**Body position and task** standing barefoot on the unstable platform, focus on the visual feedback screen in front and attempt to maintain the cursor in the centre of the screen: • measure at level 8 • measure at level 7	sign. higher mean OA, AP and ML scores in 3 vs 2 T; no sign. difference in the ML between the stability level 8 and 7 during 2 or 3 T.
**Moreira et. al. (2017) [37]**	to investigate the control of upright quiet standing throughout pregnancy;	**Static stability** COP metrics AP/ ML COP displacements: • root mean square (RMS) [mm], • mean velocity (MV) [mm/s] • 80% power frequency (f80) [Hz] • COP area [mm]	**2 triaxial force plates** (OR6, AMTI, USA, 100 Hz) synchronized with EMG	**Body position and task** upright standing position with each foot on each force plate (feet apart by ~20 cm) and arms along the body. Constant foot position: • EO: focus on a target ~2 m in front of her; • EC; • without vision. Resting period: 2 min between each trial **No of trials: 3** **Trial time:** 60 s	higher postural oscillations in AP while ML sway reduced in 3T sign. difference for EO—for all but one measure (f80 from ML COP). differences between group types detected for: f80 from ML COP; RMS from AP COP and MV from ML COP.
**Opala et al. (2018)** **[50]**	to verify the influence of the evaluation period on AP postural sway mean velocity to determine whether the Finger Floor Distance (FFD) was associated with AP postural sway	**Static stability:** CoP AP velocity [mm/s] **Trunk mobility:** FFD test value [cm]	**Force platform** (model 9281C, Kistler Instruments Corp, Winterthur, Switzerland) [100 samples/s] **35-cm-high platform** for FFD test	**Body position and tasks:** **(1) Posturographic test** Barefoot standing with the arms at sides and at a comfortable stance on a stable force platform, looking straight ahead at a wall 3m away. **Number of trials:** 2 with short break in between **Trial time:** 30 s **(2) Finger Floor Distance (FFD)** standing barefoot by the edge of a 35-cm-high platform with the feet hip-width apart and bend forward to reach the ground with fingers while keeping knees extended.	non-sign. effect of the evaluation period on CoP AP vel; a negative correlation between FFD test values and AP COP velocity at 6 m. PP,
**Danna-Dos-Santos et al. (2018) [38]**	how pregnancy affects postural control in each trimester of pregnancy	**Static stability:** • COP_ap_ and COP_ml_, computed using Fz, Mx and My recordings. • averaged position of the COP_ap_ (COP_ap_avg_), COP path area (Area), body sway range (Range_ap_ and Range_ml_), body sway mean velocity (MV_ap_ and MV_ml_), max frequency containing 80% of the COP signal power spectral densities in each direction (F80_ap_ and F80_ml_), and sample entropy estimates of the COP (SEnt_ap_, SEnt_ml_, and CrossSEnt)	**force platform** (Biomec, EMG Systems Brasil Inc.) [100 Hz]	**Body position and task:** **(1) Posturography** Participants were standing barefoot on the force platform with their feet in parallel and 10 cm apart. Upper limbs were crossed against their chest and vision directed to a physical static point placed at their eyes high and at a distance of approx. 1 m **Trial time:** 120 s	sign. increase in Area and Range_ap_ and Range_ml_ for 1T, 2T, 3T vs NP. sign. decrease of COP_ap_avg_in 3T vs NP and 1T. sign. decrease in F80_ml_, SEnt_ml_, and CrossSEnt in 1T, 2T, and 3T vs NP. no effect of pregnancy for the postural indices MV_ap_, MV_ml_, F80_ap_, SEnt_ap_.
**Takeda et al. (2018) [46]**	to clarify the posture control characteristics of women that fall during pregnancy	**Static stability** 1) **rectangular area**; 2) **COG movements** AP & ML directions; 3) **stability limits** obtained by multiplying the movement of COG between the AP and ML; • **interviews** based on medical exam. in 2, 3 T	**2 stabilometers** (JK-101 II; Unimec Co., Tokyo) + personal computer (quantitative evaluation) **self-administered questionnaire** (qualitative evaluation of the situation of falls)	**Body position and task** Subject stood barefoot on the 2 stabilometers, with medial malleoli 100 mm apart; • maintained the same posture for 10 s; • then maintained the stable posture while COG was moved as far as possible forward; • then COG was moved backward; • then to the right, • then to the left. **No of trial:** 2 **Trial time:** 10 s	Fall group: • larger COG area in the back, left and right from 2T; • sign. smaller stability limits in 3T vs non-fallers; • sign. interaction between AP COG movement; • sign. interaction with the lateral CoG movement, the main effect of time, and the main effect of experiencing a fall; • sign. interaction in the stability limits and the main effect of time, and main effect of fall
**Shingala et al. (2019) [39]**	to evaluate postural balance in 3T pregnant females using Four square step test	**Dynamic balance** Four square step test (FSST) -the ability to step quickly in different directions	**4 Canes** (< 2.5 cm in diameter), **stop-watch**	**Body position and task** Subjects both feet contact with the floor in each square and she has to face forward during the entire sequence. **No of trials:** 2 **Trial time:** mean time [s] taken as the score	sign. decrease in postural balance in 3T vs NP due to increase in time taken to complete FSST
**Sancar et al. (2021) [44]**	to examine the changes in static balance during the three trim. of pregnancy	**Static balance** Overall Stability Index (OSI), Medial-Lateral Stability Index (MLSI), Anterior-Posterior Stability Index (APSI) Sway index scores (SI-Sway Index) for: 1.EO Firm Surface, 2.EC Firm Surface, 3.EO Foam Surface, 4.EC Foam Surface	**Biodex-BioSwayTM Balance System** (SD 950–340, Biodex Medical Systems, Inc., Shirley, NY, USA)	**Body position and task** Postural Stability Test: maintaining CoG (dot on the screen) by moving the body (without moving feet). **No of trials:** 3 **Trial time:** 30 s Limits of Stability Test: woman moved the cursor representing her CoG to the targets on the screen. By shifting her weight, she had to reach the blinking target and back as quickly and with as little deviation as possible. **No of trials**: 3 Modified Clinical Test of Sensory Integration and Balance (mCTSIB): 1. EO standing on the firm surface 2. The same with EC. 3. EO standing on the foam surface placed on the platform. 4. The same with EC. **Test time**: 30 s each + 10 s rest in between	no sign. difference between trimesters in terms of the postural stability test (p>0.05); sign. increase in the LOS in 3T compared to 1T (p<0.05); sign. difference in mCTSIB in EC firm surface parameter: higher oscillations in 3T vs 2T (p<0.05)
**Ramachandra et al. (2023) [51]**	to compare the postural sway between pregnant and non-pregnant women during eight different sensory conditions including those in which vision, proprioception, and base of support are compromised	**Static stability** • Antero-posterior sway velocity (mm/s) • Medio-lateral sway velocity (mm/s) • Velocity moment (mm2/s) **The preferred stance width [cm]** measured using a measuring tape between the right and the left medial malleoli.	**Posturography system:** **force platform** (width 800 mm, height 70 mm (Good balance System, Metitur, Finland, 2007) **3-channel DC (Direct Coupled) amplifier, an 8-channel** **12byte analog to digital converter** [50 Hz], **computer program** (Good Balance software Metitur Ltd)	**Body position and task** bipedal barefoot standing position on the platform with feet parallel and arms crossed against chest and heels aligned at a reference line. Participants gaze at a black circle(12 cm diameter)on a white background fixed at a distance of 1 m at the participants’ eye level **Eight sensory conditions in randomized order:** • EO/EC standing on the firm surface (preferred stance width) • EO/EC standing on a firm surface(with feet together) • EO/EC standing on a foam surface (preferred stance width) • EO/EC standing on a foam surface(with feet together) **No of trials:** 3 trails each condition (mean of the trials–final reading) 2 min break in between the conditions **Trial time:** 30 s(recorded after 5 s of positioning of the participant on the force platform)	sign. larger stance width of compared to NP women (p < 0.001); sign. increased velocity moment and AP sway velocity compared to the CG in all eight sensory conditions(p < 0.05); ML stability in P was comparable to NP in all sensory conditions except when both groups were matched for stance width sign. difference in ML sway velocity in EO and EC feet apart condition on the firm surface(p < 0.05).

Right (R), Left (L), antero-posterior (AP) and medio-lateral (ML), trials with eyes closed (EC condition); trials with eyes open (EO condition); maximum voluntary contractions (MVCs), months–m., weeks–w., pregnancy–p., Motor Control Test–MCT, 1,2,3T –first, second and third trimester of pregnancy, P–pregnant women, NP–non pregnant, CG–control group, PF–pregnant fallers, PNF–pregnant non-fallers

### 3.5. Outcome measures and equipment applied

#### 3.5.1. Static stability

In the majority of cases, control of posture in a quiet stance was quantified by the center of pressure (COP) changes in the anterior-posterior (AP), medial-lateral (ML), or combined radial (RAD) directions from a single force platform [27, 29, 32, 35, 37, 38, 40, 48–51]. In two studies, two force platforms were used for each lower limb [37, 45]. Takeda [45] assessed stability while performing the functional reach test (FRT) from a static standing posture using two force plates for each lower limb and a 3D motion analysis system. He evaluated the maximum FRT distance [cm], COP anterior displacement at FRT max [cm], GRF at FRT max [N/kg], leg joint moments at FRT max [Nm/m/kg], and leg and trunk angles in the sagittal plane at FRT max [deg]. Then, in Takeda et al. [46], two stabilometers were used to register COG movements in AP and ML directions. In the study by Ribas & Guirro [52], a pressure platform measuring both plantar pressure and postural balance based on center of force (COF) oscillations was applied. Mean COP sway velocity (Vel) was assessed by Moreira et al., Danna-Dos-Santos et al., and Opala-Berdzik et al. [37, 38, 48]. Other measures of balance were provided by Yoo et al. [36], who assessed the weight distribution index (WDI) using four force plates of the Tetrax^®^ system. Yu et al. [49] carried out detrended fluctuation analysis (DFA), including α (an index of long-range autocorrelation in the data), to measure the positional variability of the COP in the AP and ML axes [cm]. Nagai et al. [35] analyzed the path length [cm] and area [cm^2^] of the body COP sway. Finally, Sancar et al. [44] evaluated static balance with the Biodex-BioSway TM Balance System (BBS). It was performed with the Postural Stability Test, Limits of Stability (LOS), and Modified Clinical Test of Sensory Integration and Balance (mCTSIB). As a result of the Postural Stability test, the Overall Stability Index (OSI), Medial-Lateral Stability Index (MLSI), and Anterior-Posterior Stability Index (APSI) were obtained, indicating the amount of deviation from the AP and ML axes. The patient’s score on this test assesses deviations from the center. Then, from the LOS test, defined as the maximum angle a person’s body can achieve from vertical without losing balance, the "overall" score of the individual was obtained. The mCTSIB test was used to evaluate the standing balance in different situations that the individual may encounter in daily life. As a result of the mCTSIB test, body sway was calculated, and the sway index scores (SI-Sway Index) for Eyes Open Firm Surface, Eyes Closed Firm Surface, Eyes Open Foam Surface, and Eyes Closed Foam Surface were obtained.

#### 3.5.2. Dynamic stability

As far as dynamic stability measures are concerned, we found different approaches. McCrory et al. [30, 42] and Ersal et al. [43] defined dynamic postural stability by the response to anterior and posterior translation perturbations of different magnitudes. In these studies, the Equitest platform was used to directly register COP metrics. Additionally, Ersal et al. [43] analyzed COG [cm] and Peak COP–COG [cm]. For the theoretical part of the study, Ersal et al. [43] implemented a two-segmented mathematical model to represent the dynamics of the body. The model identified the dominant strategy of stability in the subjects. Stability indices (Overall Stability Index (OA), AP Stability Index (APSI), ML Stability Index (MLSI)) were provided by the Balance platform at level 8 [41] and at levels 7 and 8 [47]. In two studies, gait trials were considered as examples of dynamic stability. Mocellin & Driusso [40] applied two force plates to measure time and the vertical and horizontal components of GRF, while Yoo et al. [36] measured gait velocity [cm/s] and cadence [steps/min] using the GAITRite system. Finally, Shingala et al. [39] applied four canes (<2.5 cm in diameter) to assess postural balance while performing the Four Square Step Test (FSST) to measure the ability to step quickly in different directions.

### 3.6. Results of syntheses

#### 3.6.1. Static stability

Most studies focusing on the static stability of pregnant women were conducted using parameters obtained by analyzing the center of pressure (COP) trajectory in both the medial-lateral (ML) and anterior-posterior (AP) directions [53]. Generally, the authors observed either a deterioration of postural stability in the sagittal plane over the course of pregnancy, no changes in COP sway along the ML direction (or even reduced sway in the third trimester), or no differences between groups. Some studies additionally considered the effects of visual condition (eyes open/closed) and support base configuration (wide/narrow) on the COP area. Most authors observed lower COP displacement areas with eyes open and feet apart, and higher values with eyes closed and feet together. Finally, in a small number of studies, COP sway was found to correlate with feelings of instability during standing, trunk flexion flexibility, and anxiety in pregnant and postpartum women [53].

In the latest review on static balance during pregnancy, the general observations were confirmed, noting significantly greater COP (AP) amplitude in the third trimester compared to the first, but no difference in COP (ML) amplitude [53]. Moreover, they concluded that there is a positive and significant correlation between AP COP oscillations and support base in women in the first trimester. Since Goossens et al. [53] did not consider four papers related to static stability included in our review, we would like to briefly mention the results of these studies. Ribas & Guirro [52] observed significantly greater COP (AP) amplitude in late pregnancy compared to early gestation, with no difference in COP (ML) amplitude. Yu et al. [49] assessed the temporal dynamics of sway and found that pregnant women with morning sickness had reduced positional variability of COP but reported greater perceived instability compared to those without morning sickness. This suggests that women with morning sickness may attempt to stabilize their bodies by reducing overall body sway, resulting in better postural stability despite feeling more unstable. Sancar et al. [44] found no significant change in postural stability throughout pregnancy. However, significant changes were detected in the last trimester in terms of sway tested in the Limits of Stability (LOS) and the Modified Clinical Test of Sensory Integration and Balance (mCTSIB). A significant increase was found in LOS in the last trimester compared to the first. According to the mCTSIB, oscillations were higher in the third trimester than in the second. Ramachandra et al. [51] pregnant women demonstrated larger median velocity moments and mean AP sway velocity compared to nonpregnant women across all tested sensory conditions. Although ML sway velocity did not show any statistically significant difference, ANCOVA results suggested a significant difference in mediolateral sway velocity in the eyes-open feet-apart condition on a firm surface and the eyes-closed feet-apart condition on a firm surface between pregnant and nonpregnant women. There was a larger velocity moment and anteroposterior postural sway velocity in pregnant women in their third trimester compared to nonpregnant women when exposed to different sensory conditions.

#### 3.6.2. Dynamic stability

What distinguishes static stability from dynamic stability is the fact that in the latter case, both the base of support (BOS) and the center of mass (COM) are in motion. Regarding dynamic stability evaluation, the authors focused on postural responses recorded via the center of pressure (COP) from underfoot force plates in different conditions, mostly providing disturbances while standing on a movable platform, then executing some balance tests, and performing overground gait trials. The results achieved were contradictory: generally showing either a decrease in postural equilibrium with pregnancy advancement, particularly in the third trimester, or an improvement of balance in late pregnancy. Let us briefly describe the main outcomes.

McCrory et al. [30] observed significantly smaller magnitude and velocity responses to perturbations during the third trimester compared to the second trimester or when compared with the control subjects (p < 0.05). While it may be argued that dynamic stability was improved in the third trimester, the authors suggest that the reduction of sway could also indicate a relative increase in torso rigidity leading to a greater risk of falling. In their second study [42], where they divided gravidas into those who experienced falls during pregnancy and those who did not, no differences were found between the nonpregnant control women and pregnant females who did not report a fall in any of the four dynamic stability variables. However, pregnant fallers demonstrated significantly less movement responses (i.e., sway velocity, and total sway) than pregnant non-fallers and the control group (p < 0.001). This could reflect an altered control system where ankle plantar flexors and dorsiflexors do not appropriately respond to control ankle joint torques.

Ersal et al. [43] used the scalar difference between the center of pressure and center of gravity (COP–COG) as a metric to characterize postural control and margin of stability assessment. Their experimental data indicated that pregnant fallers had significantly smaller peak COP–COG values compared with pregnant non-fallers and controls (p < 0.01), which may reflect the inability to generate adequate corrective torque in response to surface perturbations. This interpretation is in line with theoretical results indicating that pregnant non-fallers had higher ankle stiffness compared with pregnant fallers and controls, suggesting that ankle stiffness itself may be the dominant reason for the different dynamic response characteristics observed. The authors concluded that increasing ankle stiffness could be an important strategy to prevent falling by pregnant women.

Inanir [41] observed a decrease in postural equilibrium, particularly in the third trimester. All postural stability and fall risk test scores were significantly higher in the third trimester compared to the control group (p < 0.05). The third trimester indices for overall and medial-lateral stability index were significantly higher than the first and control groups (p = 0.001). Anterior-posterior stability index was greater in the third trimester compared to the control group. Additionally, the third-trimester fall risk test was significantly higher than in all the other groups (p < 0.001). The authors emphasized that dynamic postural stability indices may be used to predict measurements of postural equilibrium during pregnancy and thus evaluate the risk of falling.

El Shamy et al. [47] used the Biodex Balance System (BBS) at levels 7 and 8 and, similar to Inanir et al. [41], observed a decreased postural equilibrium in the third trimester compared with the second trimester of pregnancy. At both stability levels, the mean overall, anterior-posterior, and medial-lateral scores were significantly higher in the third trimester compared to the second trimester (p < 0.05). However, when comparing the scores between stability levels 8 and 7, there was no significant difference (p > 0.05) in the medial-lateral direction between the second and third trimesters of pregnancy.

Surprisingly, the results by Yoo et al. [36] revealed that the balance of pregnant women (both eyes open and eyes closed) increased toward the third trimester of pregnancy due to a significant decrease (p < 0.05) in the weight distribution index (WDI). However, WDI scores measured using the Tetrax system in pregnant women were still lower than in the control group. This means that the balance of pregnant women in the third trimester is better than that in the second trimester but similar to that of nonpregnant women. Additionally, this study revealed that the gait speed of the gravidas was significantly (p < 0.05) reduced (from 113 cm/s in the second trimester to 102 cm/s in the third trimester) compared to nonpregnant women (125 cm/s). Also, the cadence of the gravidas in the second and third trimesters (109 steps/min and 98 steps/min, respectively) showed a significant decrease (p < 0.05) when compared to nonpregnant women (114 steps/min). These findings are consistent with the results of Moccellin & Driuso [40], who registered ground reaction forces in pregnant and control groups and found that women in the first trimester and throughout pregnancy presented a trend of decreasing levels of the peaks, representing a decrease in gait velocity.

Shingala et al. [39] observed a significant decrease in postural balance in the third trimester compared to the nonpregnant group due to an increase in the time taken to complete the Four Square Step Test (FSST) (p < 0.05). For the pregnant females, they registered 13.15 ± 0.81 s (mean ± SD), while for the controls. 8.45 + 0.82 s. It showed that pregnant females took longer time to complete the test suggesting that their postural balance is compromised when compared to the control group.

## 4. Discussion

### 4.1. Principal findings

Postural stability is essential for functional independence in the pregnant population. As our study revealed, the postural control of gravid women in static conditions generally changed significantly throughout pregnancy only when visual cues were limited or the area of support was reduced. However, a few studies reported no changes in postural alignment. Importantly, research results indicate that women in advanced pregnancy may be at increased risk of falling when their vision is compromised. The results of research on dynamic stability are also inconclusive, with studies observing either a decrease in postural equilibrium with pregnancy advancement, particularly in the third trimester, or an improvement in balance in late pregnancy.

We should not be surprised by such unequivocal results, considering the complexity of integrating physiological mechanisms, processing sensory information in accordance with the postural body scheme during both standing and movement, the goals of the subjects, and their previous experience [3].

### 4.2. Larger oscillations of COP—what does it mean?

In terms of static and dynamic stability, researchers can be divided into two groups: those who recognize larger oscillations of COP as a tendency toward instability (due to factors like increased joint laxity and unevenly distributed mass around the body) [29, 32, 40, 52], and those who consider the postural control system adaptive to changes that occur during pregnancy and postpartum (mostly as changes in AP posture alignment and wider stance area) [48].

Supporters of the first interpretation suggest that, in terms of static stability, the increase of AP COP oscillations for pregnant women in the third trimester is due to increased ligament laxity [25, 54–56], which can cause greater ankle joint instability. Another hypothesis concerns the activity of the soleus muscle [37]. As revealed in the study by Moreira et al. [37], soleus EMG was not statistically different between control and pregnant groups (a similar proportion of motor neurons was recruited in both groups). Given that increased mass in pregnant women enhances the toppling torque, together with similar soleus activity, it would result in a more unstable system in the AP direction [57]. However, this hypothesis requires further exploration.

In dynamic conditions, Ersal et al. [43] identified an increase in ankle stiffness in pregnant non-fallers, which could be a response to pregnancy-related changes such as increased mass and ligament laxity [58, 59], or decreased nerve conduction velocity [60] and neuromuscular coordination [29, 35] during gestation. On the other hand, McCrory et al. [30] found alterations to dynamic stability in response to a translation postural perturbation in the third trimester: while reaction time was not affected by pregnancy, the amount of sway following the perturbation was reduced. They consider the relative stiffness of the torso to be the underlying cause. Wu et al. [61] speculated that increasing rigidity of the torso could be related to the higher incidence of falls in pregnant women. Another study by McCrory et al. [42] seems to confirm this view, showing that women who had not fallen demonstrated similar COP movement patterns during translational perturbations to nonpregnant women. However, pregnant women who reported falling presented decreased movement of the COP. This could reflect an altered control system, resulting in greater COM movement and potentially leading to a fall. The altered response to perturbation in pregnant fallers may also result from factors not assessed in this study, such as muscle strength.

As mentioned above, some authors consider the alterations in the postural control system as an adaptation to the changes that occur during pregnancy. To provide stability while standing, a pregnant woman adapts her posture by increasing lumbar lordosis and slightly tilting her body posteriorly [62]. Whitcome et al. [26] revealed that gravidas self-positioned in the natural stance maintain an almost constant center of mass position. Opala–Berdzik et al. [48] used this explanation for their results, which demonstrated unchanged AP static stability during the perinatal period, suggesting that in healthy women, the postural control system adapts to the changes that occur during pregnancy and postpartum.

At the same time, lateral stability is preserved throughout pregnancy because of the adaptive increase in stance width, which improves lateral balance [27, 35, 63]. The reason for the lack of lateral sway changes may be that a pregnant woman’s body shape changes evenly in the frontal plane, and the increasing mass is more equally distributed compared to the sagittal plane [48]. Other possible factors explaining higher stability in the ML direction are increased lumbar muscle activity [37] or the enlargement of the pelvis during pregnancy [27].

Besides bodily changes, behavioral factors may be relevant, as fear of falling can increase levels of caution in pregnant women, influencing their gait patterns. This effort to maintain equilibrium can provoke changes in the walking patterns of pregnant women [64]. One strategy used by pregnant women to maintain stability in both static and dynamic postures is repositioning their feet on the ground to increase their bases of support [62, 65–67]. Furthermore, Moccelin & Driuso [40] found that pregnant women tried to maximize their postural stability and control of sideways movements by adjusting their step width. This strategy requires adopting walking patterns that produce changes in the joint segments and lower limb muscles. Moccelin & Driuso [40] found a trend toward reduced postural control during the first trimester of pregnancy, with a further reduction by the third trimester, although no significant difference was found. Compared to the control group, pregnant women had larger COP displacement areas, longer time in the first phase of weight acceptance (the first task of the stance phase during the gait cycle, which comprises three functional demands: shock absorption, initial limb stability, and the preservation of progression [68]), lower values of the first and second peaks of the vertical component, and lower maximum and minimum values of the AP horizontal component of the GRF, possibly indicating a reduction in gait speed and thrust in the terminal stance phase of the gait cycle.

Data from Yu et al. [49] suggest that the effects of visual tasks on postural sway in the AP axis and stance width on postural sway in the ML axis can be seen during the first trimester of pregnancy. Like Jang et al. [27], they found effects on both the magnitude and dynamics of postural sway. Variations in visual tasks influenced both the magnitude of sway, as reflected in the positional variability of the COP, and the temporal dynamics of sway, as revealed by detrended fluctuation analysis of the COP data. Manipulation of stance width also influenced both the magnitude and dynamics of sway [49]. An increased amount of variability has been reported as a predictor of the risk of falling, with the assumption that it equates to increased instability. On the other hand, some evidence shows that increased variability may not be synonymous with dysfunction (i.e., postural stability decrease), implying that a moving system (e.g., a swaying body during posture or a moving body during gait) with large variability does not necessarily indicate either a highly stable system or poor stability [69].

### 4.3. Clinical applications

Health education programs are expected to be provided to future mothers so that women can improve their knowledge, attitudes, and skills for a healthy pregnancy and delivery. The above findings can be used as basic data for health promotion programs aimed at maintaining sound daily activities in pregnant women.

### 4.4. Recommendations for physical activity for pregnant women

Although exercise is safe for both the mother and fetus, most women reduce their activity level during the first weeks of gestation [70]. Therefore, professionals should encourage women to initiate or continue exercising during a healthy pregnancy. They should be more aware of the benefits of postural training aimed at improving their body stability during pregnancy and the postpartum period [27, 32, 46, 49, 52]. Lateral stability is maintained during pregnancy, likely accomplished by increasing stance width; thus, exercises employing various sizes of support bases and different visual stimuli could serve as effective interventions to minimize the effects of gestation on the control of posture while standing [32]. Takeda et al. [45] emphasized educating pregnant women that balance ability decreases from the second to the third trimester. Considering the effective interventions in the elderly population to improve postural stability, Jang et al. [27] suggest dynamic and static balance training exercises, including Tai Chi and strength training. Based on findings by Opala-Berdzik et al. [50], postpartum women, at least up to 6 months after delivery, should follow exercise recommendations similar to those for pregnant women, particularly avoiding excessive stretching that predisposes them to joint hypermobility. Given the different proprioception and kinesthetic feedback from looser connective tissue structures resulting in altered postural control, they recommend exercises that increase pelvis-spine complex stability in postpartum women. Sensory information from mechanoreceptors on the foot soles is an important part of postural control in quiet standing [71]. The study by Ramachandra et al. [72] suggests that ankle proprioception is significantly affected in the third trimester and does not return to baseline even at six weeks postpartum. This could be due to altered proprioceptive input from lax ligaments around the ankle joint and possibly mild edema, more common during the third trimester. Therefore, Ramachandra et al. [72] recommend that lower limb joint proprioceptive training, especially for the ankle joint, should be part of antenatal and postnatal exercises. Shingala et al. [39] propose a balance exercise program that can be added to the Ante-Natal program for pregnant women, with proper assessment and supervision. Takeda et al. [46] suggest employing Whipple’s [73] recommendations, emphasizing effective posture control training features: 1) body-weight exercises; 2) interactions between the body and head (eyeball movement), including quick horizontal movements; and 3) activation of muscle groups, including amplitude motion in the vertical direction and the thigh and hip joints. Additionally, Takeda et al. [46] recommend incorporating anti-gravity movements to promote muscle activity supporting body weight, being conscious of “wobble” in all directions due to weight shifts. Recognizing displacement due to changes in weight and the body’s center of gravity as pregnancy progresses is important. McCrory et al. [42], who observed that all sedentary pregnant women in their study fell during their pregnancies, suggest that exercise may play a role in fall prevention in pregnant women. Moccellin & Driuso [40] and Sancar et al. [44] encourage therapists to directly address the postural control of pregnant women and provide physical activity programs aimed at improving balance, maintaining muscle tone and strength, preventing falls, and promoting physical well-being during pregnancy. They should also educate pregnant women about pain relief and correct movement methods to help them cope with physical and functional changes due to pregnancy [36].

### 4.5. Methodological appraisal of the included studies: Strengths and limitations

We have identified some areas of weakness that should be addressed in future studies:

#### 4.5.1. Sample details

Information about the sample groups should be more detailed, including:

The recruitment process of participants, which could indicate to what extent the sample is representative and whether the findings can be generalized.

The process of participant exclusion (how many and when they were excluded), which could potentially explain the small sample sizes in some studies.

#### 4.5.2. Lack of homogeneity in study groups

The number of subjects varied across studies, with some studies having small sample sizes (<20).

The division into trimesters varied between studies (assignment to a specific trimester based on the week of pregnancy). Additionally, some studies lacked information about the week of pregnancy when the data were collected.

The lack of homogeneity in study groups may partially explain the inconclusive research results. Inconsistencies in trimester division create difficulties and reduce the credibility of comparative analysis, as women described to be at the same stage of pregnancy are assigned to trimesters according to different criteria.

#### 4.5.3. Limited longitudinal analyses

Among the analyzed studies, only six included longitudinal analysis of postural stability throughout the three trimesters of pregnancy. Longitudinal studies are demanding, requiring participants to attend multiple laboratory sessions. Consequently, some authors opted out of this approach and focused only on the most advanced period of pregnancy, using nulliparous women as a control group or comparing women at various pregnancy stages at a single time point.

#### 4.5.4. Methodology and postural indices

The time duration of trials varied greatly across studies, and only one study [38] met the Ruhe et al. [74] recommendation of ≥90 seconds. Longer recording times are necessary to observe the development of certain postural behaviors in bipedal standing [75]. Short trial times may not allow enough time to observe the actual postural strategies of individuals.

Including multiple indices of postural sway is beneficial for identifying clearer patterns of postural behavior. Although many parameters were included in the analyzed studies, few were repeatable, making comparisons between studies difficult. The equipment used also varied greatly.

The importance of stance width for stability measures has been shown; however, some studies did not include information about it (preferred/fixed stance width) in the body position description [29, 30, 40, 41].

#### 4.5.5. Precautions related to pregnancy

Due to precautions related to pregnancy, the perturbations and tasks used while testing dynamic stability may be too small to induce sufficient instability to cause stepping or loss of balance [30, 42].

Strengths of the Analyzed Studies:

1. Examination of direction-specific balance measures [27].
2. The majority of the studies (14) included information about parity. One study also included information about gravidity and showed that the groups were matched for parity/gravidity [41].
3. A few studies used a longitudinal design, allowing observation of the dynamics of changes during pregnancy.
4. Most of the papers provided not only a diagnosis of the state but also an explanation of the results achieved.

### 4.6. Strengths and limitations

The primary strength of our review lies in the identification of specific methodological weaknesses within the analyzed studies. Addressing these weaknesses in future research may lead to improved research designs and greater consistency in findings. Additionally, our review encompasses studies evaluating postural stability under both static and dynamic conditions, thereby extending the existing body of literature [53]. We have elucidated the strategies employed by pregnant females to enhance body stability, which adds a significant contribution to understanding postural adaptations during pregnancy. Importantly, we have provided recommendations and clinical implications derived from these studies aimed at reducing the risk of falls in the pregnant population. Moreover, we emphasize the limitations of the quiet stance paradigm in evaluating postural sway. This paradigm is limited in its ability to detect and control postural sway with reference to other behaviors. Traditional tests of quiet stance are not representative of real-world conditions, where individuals are often engaged in other activities while standing, such as reading, manual manipulation, or visual tracking. Therefore, future studies on postural stability throughout pregnancy should consider that postural control is integrated with the execution of suprapostural activities.

## Supporting information

S1 Checklist PRISMA 2020 checklist. (DOCX)

S1 Table Search strategy used in SportDiscuss with full text and MEDLINE database. (DOCX)

S2 Table Search strategy used in Health Source—Consumer Edition and Health Source: Nursing/Academic Edition and Rehabilitation & Sports Medicine Source database. (DOCX)

S3 Table Search strategy used in PubMed database. (DOCX)

S4 Table Modified downs and black checklist for quality assessment—Part 1. (DOCX)

S5 Table Modified downs and black checklist for quality assessment—Part 2. (DOCX)

S6 Table All studies identified in the literature search, including those that were excluded from the analyses with the reason. (DOCX)
