# Supplementary material for: Postural control patterns in gravid women—A systematic review
Source: PLoS One. 2024 Dec 27;19(12):e0312868. doi: 10.1371/journal.pone.0312868 (PMC11676516; doi:10.1371/journal.pone.0312868)
Supplement: S1 Table — (DOCX) [file pone.0312868.s002.docx]

| **Table S1.** Search strategy used in SportDiscuss With Full Text and MEDLINE databases | | |  |
| --- | --- | --- | --- |
| **#** | **Query** | **Results** | |
|  |  | **SportDiscuss With Full Text** | **MEDLINE** |
| S3 | S1 AND S2 | 39 | 187 |
| S2 | S2 pregnancy OR pregnant | 3,424 | 101,133 |
| S1 | “postural control” OR “postural balance” OR “postural stability” OR “body balance” | 3,405 | 6,229 |
| ***Interface:*** EBSCOhost Research Databases.  ***Search Screen:*** Advanced Search.  ***Limiters:*** Full text, English Language; Human; Female; Adult.  ***Database:*** SportDiscuss With Full Text and MEDLINE.  ***Searching Date:*** 01 September 2024. | | |  |
