# Supplementary material for: Postural control patterns in gravid women—A systematic review
Source: PLoS One. 2024 Dec 27;19(12):e0312868. doi: 10.1371/journal.pone.0312868 (PMC11676516; doi:10.1371/journal.pone.0312868)
Supplement: S2 Table — (DOCX) [file pone.0312868.s003.docx]

**Table S2.** Search strategy used in: **Health Source - Consumer Edition and Health Source: Nursing/Academic Edition  and Rehabilitation & Sports Medicine Source** databases

|  | | |  |  |
| --- | --- | --- | --- | --- |
| **#** | **Query** | **Results** | | |
|  |  | Health Source - Consumer Edition | Health Source: Nursing/Academic Edition | Rehabilitation & Sports Medicine Source |
| S3 | S1 AND S2 | 0 | 5 | 12 |
| S2 | pregnancy OR pregnant | 1,398 | 19,396 | 2,842 |
| S1 | “postural control” OR “postural balance” OR “postural stability” OR “body balance” | 6 | 1,258 | 2,836 |
| ***Interface:*** EBSCOhost Research Databases.  ***Search Screen:*** Advanced Search.  ***Limiters:*** Full text, English Language; Human; Female; Adult.  ***Database:*** Health Source - Consumer Edition and Health Source: Nursing/Academic Edition and Rehabilitation & Sports Medicine Source.  ***Searching Date:*** 01 September 2024. | | |  |  |
