# Supplementary material for: Postural control patterns in gravid women—A systematic review
Source: PLoS One. 2024 Dec 27;19(12):e0312868. doi: 10.1371/journal.pone.0312868 (PMC11676516; doi:10.1371/journal.pone.0312868)
Supplement: S3 Table — (DOCX) [file pone.0312868.s004.docx]

| **Table S3.** Search strategy used in PubMed database | | |
| --- | --- | --- |
| **#** | **Query** | **Results** |
| S3 | #1 AND #2  (("postural control"[All Fields] OR "postural balance"[All Fields] OR "postural stability"[All Fields] OR "body balance"[All Fields]) AND ("humans"[MeSH Terms] AND "female"[MeSH Terms] AND "english"[Language] AND "adult"[MeSH Terms:noexp] AND 1990/01/01:2023/12/31[Date - Publication]) AND (("pregnancy"[MeSH Terms] OR "pregnancy"[All Fields] OR "pregnancies"[All Fields] OR "pregnancy s"[All Fields] OR ("pregnant"[All Fields] OR "pregnants"[All Fields])) AND ("loattrfull text"[Filter] AND "humans"[MeSH Terms] AND "female"[MeSH Terms] AND "english"[Language] AND "adult"[MeSH Terms:noexp] AND 1990/01/01:2023/12/31[Date - Publication]))) AND ((fft[Filter]) AND (humans[Filter]) AND (female[Filter]) AND (english[Filter]) AND (adult[Filter])) | 56 |
| S2 | ("pregnancy"[MeSH Terms] OR "pregnancy"[All Fields] OR "pregnancies"[All Fields] OR "pregnancy s"[All Fields] OR "pregnant"[All Fields] OR "pregnants"[All Fields]) AND ((fft[Filter]) AND (humans[Filter]) AND (female[Filter]) AND (english[Filter]) AND (adult[Filter]) AND (1990:2023[pdat])) | 217,968 |
| S1 | "postural control"[All Fields] OR "postural balance"[All Fields] OR "postural stability"[All Fields] OR "body balance"[All Fields]) AND ((humans[Filter]) AND (female[Filter]) AND (english[Filter]) AND (adult[Filter]) AND (1990:2023[pdat])) | 8,233 |
| ***Interface:*** National Library of Medicine.  ***Search Screen:*** Advanced Search.  ***Filters:*** Full text, English Language; Humans; Female; Adult.  ***Database:*** PubMed.  ***Searching Date:*** 01. September 2024. | | |
