# Supplementary material for: Postural control patterns in gravid women—A systematic review
Source: PLoS One. 2024 Dec 27;19(12):e0312868. doi: 10.1371/journal.pone.0312868 (PMC11676516; doi:10.1371/journal.pone.0312868)
Supplement: S4 Table — (DOCX) [file pone.0312868.s005.docx]

**Table S4.** Modified Downs and Black Checklist for QualityAssessment - Part 1.

|  | **Study** | | | | | | | | | | |
| --- | --- | --- | --- | --- | --- | --- | --- | --- | --- | --- | --- |
| **ChecklictCriteria** | **Butler**  **[17]** | **Ribas [39]** | **Jang**  **[15]** | **Nagai**  **[23]** | **Oliveira [20]** | **McCrory**  **[18]** | **McCrory**  **[30]** | **Moccellin [28]** | **Yu**  **[37]** | **Inanir [29]** | **Ersal [31]** |
| REPORTING |  | | | | | | | | | | |
| Q1 **Is the hypothesis/aim/objective of the study clearly described?** | **Y** | **Y** | **Y** | **Y** | **Y** | **Y** | **Y** | **Y** | **Y** | **Y** | **Y** |
| Q2 **Are the main outcomes to be measured clearly described in the Introduction or Methods section?** | **Y** | **Y** | **Y** | **Y** | **Y** | **Y** | **Y** | **Y** | **Y** | **Y** | **Y** |
| Q3**Are the characteristics of the patients included in the study clearly described?** | **Y** | **Y** | **N/U** | **Y** | **Y** | **Y** | **Y** | **Y** | **Y** | **Y** | **N/U** |
| Q6 **Are the mainfindings of the studyclearlydescribed?** | **Y** | **Y** | **Y** | **Y** | **Y** | **Y** | **Y** | **N/U** | **Y** | **Y** | **Y** |
| Q7 **Does the study provide estimates of the random variability in the data for the main outcomes?** | **Y** | **Y** | **Y** | **Y** | **Y** | **Y** | **Y** | **Y** | **Y** | **Y** | **N/U** |
| Q9 **Have the characteristics of patients lost to follow-up been described?** | **N/U** | **N/U** | **Y** | **N/U** | **N/U** | **Y** | **Y** | **Y** | **N/U** | **N/U** | **Y** |
| Q10 **Have actual probability values been reported (e.g.0.035 rather than<0.05) for the main outcomes except where the probability value is less than 0.001?** | **N/U** | **Y** | **Y** | **Y** | **Y** | **Y** | **Y** | **Y** | **Y** | **Y** | **Y** |
| EXTERNAL VALIDITY |  | | | | | | | | | | |
| Q 12 **Were the subjectsasked to participate in the studyrepresentative of the entirepopulation from whichtheywererecruited?** | **N/U** | **N/U** | **Y** | **N/U** | **Y** | **Y** | **Y** | **Y** | **Y** | **N/U** | **N/U** |
| Q13**Were the staff, places, and facilities where the patients were treated, representative of the treatment the majority of patients receive?** | **Y** | **N/U** | **N/U** | **N/U** | **N/U** | **Y** | **Y** | **Y** | **N/U** | **Y** | **N/U** |
| INTERNAL VALIDITY - BIAS |  | | | | | | | | | | |
| Q16 **Ifany of the results of the studywerebased on “data dredging”, was thismadeclear?** | **Y** | **Y** | **Y** | **Y** | **Y** | **Y** | **Y** | **Y** | **Y** | **Y** | **Y** |
| Q 17 **In trials and cohort studies, do the analyses adjust for different lengths of follow-up of patients, or in case-control studies, is the time period between the intervention and outcome the same for cases and controls?** | **Y** | **N/U** | **Y** | **N/U** | **N/U** | **Y** | **Y** | **Y** | **N/U** | **Y** | **Y** |
| Q 18**Were the statistical tests used to assess the main outcomes appropriate?** | **Y** | **Y** | **Y** | **Y** | **Y** | **Y** | **Y** | **Y** | **Y** | **Y** | **Y** |
| INTERNAL VALIDITY – CONFOUNDING (SELECTION BIAS) |  | | | | | | | | | | |
| Q 26 **Werelosses of patients to follow-uptakenintoaccount?** | **N/U** | **N/U** | **Y** | **N/U** | **N/U** | **Y** | **Y** | **Y** | **N/U** | **N/U** | **Y** |
| TOTAL SCORE | **9** | **8** | **11** | **8** | **9** | **13** | **13** | **12** | **9** | **10** | **9** |

* Y=1 point, N/U=0 points; **Abbreviations:** N/U, no/unable to determine; Y, yes
