# Supplementary material for: Postural control patterns in gravid women—A systematic review
Source: PLoS One. 2024 Dec 27;19(12):e0312868. doi: 10.1371/journal.pone.0312868 (PMC11676516; doi:10.1371/journal.pone.0312868)
Supplement: S6 Table — (DOCX) [file pone.0312868.s007.docx]

Table S6. All studies identified in the literature search, including those that were excluded from the analyses with the reason.

| no | **Study** | **Meeting inclusion criteria /reason** |
| --- | --- | --- |
|  | Butler, E. E., Colón, I., Druzin, M. L., Rose, J. (2006). Postural equilibrium during pregnancy: decreased stability with an increased reliance on visual cues. American journal of obstetrics and gynecology, 195(4), 1104-1108. | Yes |
|  | Ribas SI., Guirro ECO. Analysis of plantar pressure and postural balance during different phases of pregnancy. Brazilian Journal of Physical Therapy 11 2007: 391-396. | Yes |
|  | Jang, J., Hsiao, K. T., & Hsiao-Wecksler, E. T. (2008). Balance (perceived and actual) and preferred stance width during pregnancy. Clinical biomechanics, 23(4), 468-476. | Yes |
|  | Nagai, M., Isida, M., Saitoh, J., Hirata, Y., Natori, H., Wada, M. (2009). Characteristics of the control of standing posture during pregnancy. Neuroscience letters, 462(2), 130-134. | Yes |
|  | Oliveira, L. F., Vieira, T. M., Macedo, A. R., Simpson, D. M., & Nadal, J. (2009). Postural sway changes during pregnancy: a descriptive study using stabilometry. European Journal of Obstetrics & Gynecology and Reproductive Biology, 147(1), 25-28. | Yes |
|  | McCrory, J. L., Chambers, A. J., Daftary, A., & Redfern, M. S. (2010). Dynamic postural stability during advancing pregnancy. Journal of biomechanics, 43(12), 2434-2439. | Yes |
|  | McCrory, J. L., Chambers, A. J., Daftary, A., & Redfern, M. S. (2010). Dynamic postural stability in pregnant fallers and non‐fallers. BJOG: An International Journal of Obstetrics & Gynaecology, 117(8), 954-962. | Yes |
|  | Moccellin, A. S., & Driusso, P. (2012). Adjustments in static and dynamic postural control during pregnancy and their relationship with quality of life: A descriptive study. Fisioterapia, 34(5), 196-202. | Yes |
|  | Yu, Y., Chung, H. C., Hemingway, L., & Stoffregen, T. A. (2013). Standing body sway in women with and without morning sickness in pregnancy. Gait & posture, 37(1), 103-107. | Yes |
|  | Inanir, A., Cakmak, B., Hisim, Y., & Demirturk, F. (2014). Evaluation of postural equilibrium and fall risk during pregnancy. Gait & posture, 39(4), 1122-1125. | Yes |
|  | Ersal, T., McCrory, J. L., & Sienko, K. H. (2014). Theoretical and experimental indicators of falls during pregnancy as assessed by postural perturbations. Gait & posture, 39(1), 218-223. | Yes |
|  | Takeda, K., Shimizu, K., & Imura, M. (2015). Changes in balance strategy in the third trimester. Journal of physical therapy science, 27(6), 1813-1817. | Yes |
|  | Yoo, H., Shin, D., & Song, C. (2015). Changes in the spinal curvature, degree of pain, balance ability, and gait ability according to pregnancy period in pregnant and nonpregnant women. Journal of physical therapy science, 27(1), 279-284. | Yes |
|  | Opala-Berdzik, A., Błaszczyk, J. W., Bacik, B., Cieślińska-Świder, J., Świder, D., Sobota, G., & Markiewicz, A. (2015). Static postural stability in women during and after pregnancy: a prospective longitudinal study. PloS one, 10(6), e0124207. | Yes |
|  | El-Shamy, F., Ghait, A., & Morsy, M. (2016). Evaluation of postural stability in pregnant women. British Journal of Medicine and Medical Research, 11(10), 1-5. | Yes |
|  | Moreira, L. S., Elias, L. A., Gomide, A. B., Vieira, M. F., & Do Amaral, W. N. (2017). A longitudinal assessment of myoelectric activity, postural sway, and low-back pain during pregnancy. Acta of bioengineering and biomechanics, 19(3), 77-83. | Yes |
|  | Opala-Berdzik, A., Błaszczyk, J. W., Świder, D., & Cieślińska-Świder, J. (2018). Trunk forward flexion mobility in reference to postural sway in women after delivery: A prospective longitudinal comparison between early pregnancy and 2-and 6-month postpartum follow-ups. Clinical Biomechanics, 56, 70-74. | Yes |
|  | Danna-Dos-Santos, A., Magalhaes, A. T., Silva, B. A., Duarte, B. S., Barros, G. L., Maria De Fátima, C. S., ... & Cardoso, V. S. (2018). Upright balance control strategies during pregnancy. Gait & posture, 66, 7-12. | Yes |
|  | Takeda, K., Yoshikata, H., & Imura, M. (2018). Changes in posture control of women that fall during pregnancy. Int J Womens Health Reprod Sci, 6(3), 255-262. | Yes |
|  | Shingala, R. K., Desai, M., Honkalas, P., & Kumar, A. (2019). Evaluation of postural balance in third trimester pregnancy. International Journal of Physiotherapy and Research, 7(4), 3109-3112. | Yes |
|  | Sancar, Ş., Atalay Güzel, N., Çobanoğlu, G., Özdemir, Y., & Bayram, M. The changes in static balance during pregnancy: a prospective longitudinal study. Clin Exp Health Sci. 2021; 11: 127. | Yes |
|  | Ramachandra, P., Kumar, P., Bø, K., & Maiya, G. A. (2023). Comparison of static postural sway characteristics between pregnant and non-pregnant women. Journal of Biomechanics, 154, 111618. | Yes |
|  | Cakmak, B., Inanir, A., & Nacar, M. C. (2015). Postural balance in pregnancies complicated by hyperemesis gravidarum. The Journal of Maternal-Fetal & Neonatal Medicine, 28(7), 819-822. | No/  no healthy pregnant females |
|  | Öztürk, G., Geler Külcü, D., Aydoğ, E., Kaspar, Ç., & Uğurel, B. (2016). Effects of lower back pain on postural equilibrium and fall risk during the third trimester of pregnancy. The Journal of Maternal-Fetal & Neonatal Medicine, 29(8), 1358-1362. | No/  no healthy pregnant females |
|  | Ojukwu, C. P., Anyanwu, E. G., & Nwafor, G. G. (2017). Correlation between Foot arch index and the intensity of foot, knee, and lower back pain among pregnant women in a South-Eastern Nigerian Community. Medical Principles and Practice, 26(5), 480-484. | No/  no healthy pregnant females |
|  | Pardo, F. J. V., Del Amo, A. L., Rios, M. P., Gijon-Nogueron, G., & Yuste, C. C. (2018). Changes in foot posture during pregnancy and their relation with musculoskeletal pain: A longitudinal cohort study. Women and Birth, 31(2), e84-e88. | No/  no healthy pregnant females |
|  | Valerio, P. M., Gonçalves, V. E., Zordão, C. C., Rezende, M. S., Moisés, E. C. D., & de Olveira Guirro, E. C. (2020). Influence of type 1 diabetes on the postural control of women in the third gestational trimester. Clinical biomechanics, 77, 105062. | No/  no healthy pregnant females |
|  | de Sousa Oliveira, G. V., Dibai Filho, A. V., Dibai, D. B., Silva, F. D. M. A. M., Firmo, W. D. C. A. et al. (2021). Correlation between baropodometric variables, disability, and intensity of low back pain in pregnant women in the third trimester. Journal of Bodywork and Movement Therapies, 25, 24-27. | No/  no healthy pregnant females |
|  | Doğan, H., & Çaltekin, M. D. (2023). Plantar sensation, proprioception, and balance levels in pregnant women with gestational diabetes mellitus. Clinical Biomechanics, 107, 106016. | No/  no healthy pregnant females |
|  | Vardi, S., Hellerstein, D., Gilleard, W., & Dunsky, A. (2017). Standing postural control during twin pregnancy: case study. young, 13, 14. | No/  multiple pregnancy |
|  | Bailey, J. F., Sparrey, C. J., Williams, F. M., Curran, P. F., Lotz, J. C., & Kramer, P. A. (2020). The effect of parity on age-related degenerative changes in sagittal balance. Spine, 45(4), E210-E216. | No/  multiple pregnancy |
|  | Cakmak, B., Inanir, A., Nacar, M. C., & Filiz, B. (2014). The effect of maternity support belts on postural balance in pregnancy. PM&R, 6(7), 624-628. | No/  intervention applied |
|  | Yan, C. F., Hung, Y. C., Gau, M. L., & Lin, K. C. (2014). Effects of a stability ball exercise programme on low back pain and daily life interference during pregnancy. Midwifery, 30(4), 412-419. | No/  intervention applied |
|  | Carver, A. R., Tamayo, E., Perez-Polo, J. R., Saade, G. R., Hankins, G. D., & Costantine, M. M. (2014). The effect of maternal pravastatin therapy on adverse sensorimotor outcomes of the offspring in a murine model of preeclampsia. International Journal of Developmental Neuroscience, 33, 33-40. | No/  intervention applied |
|  | Watelain, E., Pinti, A., Doya, R., Garnier, C., Toumi, H., & Boudet, S. (2017). Benefits of physical activities centered on the trunk for pregnant women. The Physician and Sportsmedicine, 45(3), 293-302. | No/  intervention applied |
|  | Bey, M. E., Arampatzis, A., & Legerlotz, K. (2018). The effect of a maternity support belt on static stability and posture in pregnant and non-pregnant women. Journal of biomechanics, 75, 123-128. | No/  intervention applied |
|  | Carvalho, A. F., Dufresne, S. S., De Oliveira, M. R., Furlanetto, K. C., Dubois, M. et al. (2020). Effects of lumbar stabilization and muscular stretching on pain, disabilities, postural control and muscle activation in pregnant woman with low back pain. Eur J Phys Rehabil Med, 56(3), 297-306. | No/  intervention applied |
|  | Hausselle, J., Haddox, A. G., Kasitz, J., & Azoug, A. (2021). An interventional exploratory study to assess the effect of footwear on postural stability and strategy during quiet standing. International Biomechanics, 8(1), 63-74. | No/  intervention applied |
|  | Ribeiro, A. P., João, S. M. A., & Sacco, I. C. N. (2013). Static and dynamic biomechanical adaptations of the lower limbs and gait pattern changes during pregnancy. Women’s Health, 9(1), 99-108. | No/  no original or peer reviewed article |
|  | Goossens, N., Massé-Alarie, H., Aldabe, D., Verbrugghe, J., & Janssens, L. (2022). Changes in static balance during pregnancy and postpartum: A systematic review. Gait & Posture, 96, 160-172. | No/  no original or peer reviewed article |
|  | Hrvatin, I., & Rugelj, D. (2022). Risk factors for accidental falls during pregnancy–a systematic literature review. The Journal of Maternal-Fetal & Neonatal Medicine, 35(25), 7015-7024. | No/  no original or peer reviewed article |
|  | Lefranc AS, Klute GK, Neptune RR. The Influence of Multiple Pregnancies on Gait Asymmetry: A Case Study. J Appl Biomech. 2023 Sep 13;39(6):403-413. | No/  no full text available |
|  | Shibayama, Y., Kuwata, T., Yamaguchi, J., Matsumoto, M., Watanabe, M. et al. (2016). Changes in standing body sway of pregnant women after long-term bed rest. Journal of Obstetrics and Gynaecology, 36(4), 479-482. | No/  no full text available |

2.For every excluded study, the table should list the reason(s) for exclusion.
